# Modulation of DNA methylation and phenotypic switching in Smooth Muscle Cells by the extracellular matrix microenvironment

**DOI:** 10.1186/1756-8935-6-S1-P34

**Published:** 2013-03-18

**Authors:** Jia-Xin Jiang, Karen J Aitken, Tyler P Kirwan, Nicole D Zhang, Shuye Pu, Darius J Bägli

**Affiliations:** 1 Department of Physiology, University of Toronto, Toronto, ON, Canada, M5S 1A8; 2 Department of Urology, Hospital for Sick Children, Toronto, ON, Canada, M5G 1X8; 3 Research Institute, Department of Developmental & Stem Cell Biology Hospital for Sick Children, Toronto, ON, Canada, M5G 1X8; 4 Centre for Computational Medicine, Hospital for Sick Children, Toronto, ON, Canada, M5G 1X8

## Background

Partial bladder outlet obstruction due to neurogenic bladder or mechanical obstruction is common amongst the population and can cause bladder injury and dysfunction. Bladder Smooth Muscle Cells (BSMCs) undergo phenotypic changes such as hyper-proliferation, de-differentiation and altered expression of integrins and ECM proteins.[1] Extracellular matrix changes are often crucial inciting events for fibroproliferative disease.[2] Epigenetic change, specifically DNA methylation, may be important factors underlying the persistent fibroproliferative phenotype. Previously, damaged matrix (heat-denatured collagen, DNC) induced hyper-proliferation of bladder smooth muscle cells (BSMC) and the phenotype was not reverted upon a return to normal matrix. [3] We examined the dependency of matrix-induced fibroproliferation and SMC phenotype on DNA methyltransferase activity. The cooperativity of matrix with other inciting stimuli (growth factors, hypoxia and strain) associated with bladder obstruction was also examined.

## Material and methods

Primary cultures of neonatal rat BSMC (early passage of 0-2) and human BSMC were plated on 12-, 24-well or 10cm culture plates that are pre-coated with either type I bovine native collagen (NC) or DNC (heat-denatured NC). Hypoxia was induced at 3% O_2_ and 5%CO_2_ with a balance of N_2_. Mechanical strain was applied with slow ramping up to a final 5% elongation over 16 hours. Inhibitors were added 2-3 hours after plating and remained for 48hrs. Cells were fixed, stained for DNA methyltransferase 3A (DNMT3A), α-Smooth muscle actin (α-SMA) and other SMC differentiation markers. Intensities and cell numbers were analysed on ImageJ Finally, Illumina 450K array of CpG sites was performed on bisulfite converted DNA from human smooth muscle cells on DNC vs. NC.

## Results

DNC exposure significantly increased the translocalization of DNMT3A into the nucleus of BSMC, in contrast to NC cells, which expressed only cytoplasmic DNMT3A. The increase in nuclear expression of DNMT3A was coupled with decreased expression level of α-SMA. Hypoxia with DNC increased DNMT3A nuclear expression, but mechanical strain only mildly increased DNMT3A expression. On DNC cultures, AG490 (JAK2/STAT inhibitor) significantly reduced DNMT3A nuclear localization (P=0.001), without changing α-SMA expression and proliferation. Aza-cytidine suppressed hyper-proliferation of BSMCs cultured on DNC without affecting basal proliferation on NC. On damaged matrix, Sonic hedgehog (SHH) upregulated expression of α-SMA, but did not alter proliferation. However, SHH increased absolute levels of both nuclear and cytoplasmic DNMT3A. Followed by rigorous multiple testing, we discovered significant changes in methylation status for 7 genes in the Illumina 450K array.

## Conclusions

Matrix exquisitely regulates DNMT3A localization and expression, and influences differentiation in BSMCs exposed to denatured matrix +/- growth factors or SHH. That nuclear expression of DNMT does not always correspond to decreased α-SMA expression suggests that DNA methylation may not directly act as a dedifferentiating factor, as it appears to influence both proliferation/loss of differentiation on DNC as well as differentiation by SHH. Future work will examine how expression of other SMC markers is affected by shRNA knockdown of DNMTs.
